# Editorial: Advances in the research of diabetic retinopathy, volume II

**DOI:** 10.3389/fendo.2023.1281490

**Published:** 2023-09-08

**Authors:** Mohd Imtiaz Nawaz

**Affiliations:** ^1^ Department of Ophthalmology, College of Medicine, King Saud University, Riyadh, Saudi Arabia; ^2^ Dr. Nasser Al-Rashid Research Chair in Ophthalmology, Abdulaziz University Hospital, Riyadh, Saudi Arabia

**Keywords:** diabetic retinopathy (DR), pathological complication, scanning swept source optical coherence tomography angiography (SS-OCTA), artificial intelligence (AI), risk predictive model, adjuvant effect

Diabetic retinopathy (DR) is a progressive disease of the retina. Diabetic retinopathy occurs because of long-term accumulated functional and structural impairments in the diabetic retina. The global prevalence of any DR form among diabetic patients is estimated to be around 27.0% (1). It takes several years before any clinical signs of DR appear in a diabetic patient, making it difficult to properly evaluate and diagnose the patient at an early stage of the disease. Diabetic retinopathy is a multifactorial disease arising from the complex interplay between dysregulated biochemical and metabolic pathways. Diabetic retinopathy begins as non-proliferative retinal abnormalities and progresses to proliferative diabetic retinopathy (PDR), characterized by a persistent low grade of inflammation and neovascularization (2, 3). The implication of several inflammatory pathways and angiogenesis processes complicates the pathology through the initiation of retinal neovascularization, vitreous hemorrhage, and/or tractional retinal detachment, which are hallmark features of PDR (4).

Clinically, the use of photocoagulation and vitrectomy remains the standard of care for treating severe complications of PDR. However, these treatments are either destructive or their successful implementation approaches are limited. Additional PDR treatment strategies involve surgical procedures to remove a thin epiretinal membrane from the surface of the retina, which further allows the retina to remodel and reattach. Despite dramatic developments, vitreoretinal surgery for epiretinal membranes is often dissatisfying both anatomically and functionally (5, 6).

The discovery of the role of the potential angiogenic modulator vascular endothelial growth factor (VEGF) in PDR has led to the development of anti-VEGF agents as therapies. However, limitations to anti-VEGF interventions exist that include a short duration of action, the presence of adverse side effects, and a poor response in a significant percentage of patients (7, 8). Furthermore, various pro-inflammatory and angiogenic factors other than VEGF may play a role in PDR (reviewed in (2, 9)), causing resistance to anti-VEGF interventions.

This observation, therefore, suggests that PDR-associated pathogenesis is multifactorial and that factors beyond VEGF may be playing a role in the initiation and progression of the disease. Thus, more theoretical, or clinical insight into the pathogenesis of diabetic retinopathy is warranted. More profound knowledge could help in developing novel approaches to target dysregulated molecular pathways, or increasing target affinity, and shortening treatment durability for the management of PDR.

Given the success of the first edition of the Research Topic *Advances in the Research of Diabetic Retinopathy* (10), and the continuing advancement in the field, we aimed to launch *Volume II* of the edition. The aim of *Volume II* was to seek more research articles exploring new paradigms toward understating the pathological mechanisms that are involved in early retinal vascular damage in patients with diabetic retinopathy. To meet this demand, *Volume II* of the edition was also overwhelmed by the publication of many exciting articles, including original research as well as reviews. Articles addressing or discussing new therapeutic implications for the early management of diabetic retinopathy were given equal space.

The editor of this topic strongly believes that articles published in *volume II* of the Research Topic could have added some knowledge to improve our understanding of the pathogenesis associated with diabetic retinopathy.

Likewise, the work by Su et al. reported that a genetically higher hip circumference is associated with a lower risk of DR. Serum levels of acylcarnitine 8:0 (Jin et al.) and cellular communication network factor-1 (Xiang et al.) can serve as predictive biomarkers for DR identification at an early stage of the disease. Using a confocal scanning laser ophthalmoscope, Song et al. demonstrated that the retinal branch arterial tortuosity may be a direct and specific indicator for early detection or assessment of DR severity. Recent scientific advancements in the use of scanning swept-source optical coherence tomography angiography (SS-OCTA) devices (Zeng et al., Qi et al., Xu et al., Lin et al., and Li et al.) could be an important clinical tool in assessing the early diabetes-induced changes in choroidal or retinal capillaries in DR patients. Furthermore, using OCT, Yao et al. showed preclinical DR may be more severe in diabetic nephropathy (DN) individuals in regard to microvascular and microstructural impairments. Similarly, Xiaodong et al. demonstrated that peripheral blood inflammatory biomarkers and OCT retinal macular imaging indexes have important value for risk prediction and diagnosis of DN in combination with DR. Hsieh et al. showed partial inner segment-outer segment layers are predictive of better response, whereas the presence of epiretinal membrane is a significant predictor of poor response to anti-VEGF treatment in eyes with diabetic macular edema.

Nevertheless, the use of artificial intelligence, or machine learning, and risk nomogram prediction models has been finding its place as a training aid system for assessing the degree of DR pathogenesis in type 2 diabetic patients. Accordingly, using fundus images from real-world diabetics, Qian et al. discussed the AI-based system for high diagnostic accuracy for the detection of DR. Wang et al. developed a predictive risk nomogram using retinal vascular geometry parameters and clinical information with no blood test requirements to facilitate risk stratification and early detection of DR. Independent common or potential predictors were tested to establish and validate a predictive model for DR (Yang et al., Wang et al.). Such a quick screening model can assist clinicians and researchers, based on a minimal amount of clinical data, to quickly determine if a diabetic patient is prone to developing DR.

Among the review topics discussing advancements in PDR treatment strategies, Lin et al. discussed targeted retinal photocoagulation, an emerging laser technology, in combination with anti-VEGF for the management of retinal diseases. A network meta-analysis review article by Wang et al. concluded that there are no distensible effects of intraoperative intravitreal conbercept (IVC) on PDR, but preoperative, except for very long intervals, is an effective adjuvant to par-plana vitrectomy for treating PDR. The analysis was indeed confirmed in an original article by Yang et al. showing that an IVC treatment that was administered 7 days preoperatively was associated with better effectiveness and a lower vitreous VEGF concentration than its administration at other time points.

A growing piece of evidence suggests that various pro-inflammatory and angiogenic factors, other than VEGF may play a role in the progression of pathogenesis associated with PDR. Accordingly, a review by Xu et al. highlights the therapeutic roles of pigment epithelium-derived factors and their receptors in the diagnosis and management of retinal diseases, including PDR. Lastly, two review articles report the significance of oral Chinese patent medicines in improving visual acuity and fundus lesions in non-PDR (Liu et al.) and PDR (Huai et al.) patients. However, the relevant clinical trials on the use of many such Chinese medicines are few, and more high-quality clinical trials await to determine their effectiveness and safety. Towards this, a study by Kim et al. suggests that a 60% edible ethanolic and catechin 7-O-b-Dapiofuranoside extract of *Ulmus davidiana* could be a potential therapeutic agent for reducing vascular leakage by preventing pericyte apoptosis in DR.

In conclusion, *Volume II* of the Research Topic brings new insights and novel data toward understanding the early retinal vascular damage or pathological mechanism involved in the initiation and progression of diabetic retinopathy. The pool of data obtained using an ultrawide SS-OCTA device or predictive nomogram model provides a wealth of knowledge regarding the early assessment or pathological grading of DR. A few research articles dedicated to understanding the role of potential biomarkers could open new therapeutic avenues for the early management of diabetic retinopathy. Nevertheless, the adjuvant effects of conbercept or oral Chinese medicine could be a game changer for the management of diabetic retinopathy.

The editor of this Research Topic strongly feels that this set of articles could be a benchmark and may add some clinical knowledge in the field of diabetic retinopathy. Last but not least, the editor invites more interdisciplinary research towards early assessment and development of treatment strategies for the management of diabetic retinopathy.

## Author contributions

MN: Conceptualization, Writing – original draft, Writing – review & editing.

## References

[1] Thomas RL Halim S Gurudas S Sivaprasad S Owens DR. IDF Diabetes Atlas: A review of studies utilising retinal photography on the global prevalence of diabetes related retinopathy between 2015 and 2018. Diabetes Res Clin Pract (2019) 157:107840. doi: 10.1016/j.diabres.2019.107840 31733978

[2] Semeraro F Cancarini A Dell’omo R Rezzola S Romano MR Costagliola C. Diabetic retinopathy: vascular and inflammatory disease. J Diabetes Res (2015) 2015:582060. doi: 10.1155/2015/582060 26137497 PMC4475523

[3] Capitao M Soares R. Angiogenesis and inflammation crosstalk in diabetic retinopathy. J Cell Biochem (2016) 117(11):2443–53. doi: 10.1002/jcb.25575 27128219

[4] Nawaz IM Rezzola S Cancarini A Russo A Costagliola C Semeraro F. Human vitreous in proliferative diabetic retinopathy: Characterization and translational implications. Prog Retin Eye Res (2019) 72:100756. doi: 10.1016/j.preteyeres.2019.03.002 30951889

[5] Charteris DG Sethi CS Lewis GP Fisher SK. Proliferative vitreoretinopathy-developments in adjunctive treatment and retinal pathology. Eye (Lond) (2002) 16(4):369–74. doi: 10.1038/sj.eye.6700194 12101443

[6] Pastor JC de la Rua ER Martin F. Proliferative vitreoretinopathy: risk factors and pathobiology. Prog Retin Eye Res (2002) 21(1):127–44. doi: 10.1016/S1350-9462(01)00023-4 11906814

[7] Kieran MW Kalluri R Cho YJ. The VEGF pathway in cancer and disease: responses, resistance, and the path forward. Cold Spring Harb Perspect Med (2012) 2(12):a006593. doi: 10.1101/cshperspect.a006593 23209176 PMC3543071

[8] van Wijngaarden P Qureshi SH. Inhibitors of vascular endothelial growth factor (VEGF) in the management of neovascular age-related macular degeneration: a review of current practice. Clin Exp Optometry (2008) 91(5):427–37. doi: 10.1111/j.1444-0938.2008.00305.x 18637105

[9] Urias EA Urias GA Monickaraj F Mcguire P Das A. Novel therapeutic targets in diabetic macular edema: Beyond VEGF. Vision Res (2017) 139:221–7. doi: 10.1016/j.visres.2017.06.015 28993218

[10] Chakrabarti S Lanza M Siddiqui K. Editorial: Advances in the research of diabetic retinopathy. Front Endocrinol (Lausanne) (2022) 13:1038056. doi: 10.3389/fendo.2022.1038056 36387845 PMC9641289

